# Phase Equilibria and Crystal Growth in the Alkali Antimonate Systems Sb_2_O_4_–NaSbO_3/_ Sb_2_O_4_–KSbO_3/_, and Sb_2_O_4_–NaSbO_3_–NaF*

**DOI:** 10.6028/jres.080A.070

**Published:** 1976-10-01

**Authors:** J. L. Waring, R. S. Roth, H. S. Parker, W. S. Brower

**Affiliations:** Institute for Materials Research, National Bureau of Standards, Washington, D.C. 20234

**Keywords:** Alkali antimonates, crystal growth, ionic conductors, potassium antimonate, sodium antimonate, sodium antimony oxyfluoride

## Abstract

Phase equilibrium diagrams have been constructed from experimental data for the systems Sb_2_O_4_–NaSbO_3_, Sb_2_O_4_–KSbO_3_, and Sb_2_O_4_–NaSbO_3_–NaF. The system Sb_2_O_4_–NaSbO_3_ contains only an intermediate pyrochlore type solid solution with a maximum melting point of 1490 °C at a Na:Sb atom ratio of 3:5. The Sb_2_O_4_–KSbO_3_ system contains in addition to the pyrochlore phase a compound 3K_2_O • 5Sb_2_O_5_ which melts congruently at about 1450 °C and two polymorphs of K_2_O • 2Sb_2_O_5_. The low temperature form of K_2_O • 2Sb_2_O_5_ was found to be monoclinic P2_1_/c with *a* = 7.178, *b* = 13.378, *c* = 11.985 A, *β* = 124°10′. The melting point of Sb_2_O_4_ was found to be 1350 *±* 5 °C and NaSbO_3_ and KSbO_3_ both melt congruently at 1555 ± 5 °C and 1410 ± 5 °C respectively. The previously reported cubic form of KSbO_3_ was found to be a K^+^ deficient phase stabilized by reaction with atmospheric moisture. A similar cubic phase which appears to be a good Na^+^ ion conductor can be synthesized in the ternary system NaSbO_3_–Sb_2_O_4_–NaF.

## 1. Introduction

The search for potential candidates for ionic conductors together with the lack of detailed phase equilibrium data has served as an impetus to investigate the alkali antimonate systems.

In the system Na_2_O–Sb_2_O_4_–O_2_ the compound NaSbO_3_ was reported to occur by Schrewelius [1]^1^ and to be hexagonal with an ilmenite structure, *a* = 5.316, *c* = 15.95 A. A pyrochlore solid solution was found to occur by Steward and Knop [2]. No melting data was previously reported.

In the system K_2_O–Sb_2_O_4_–O_2_ the compound KSbO_3_ with an ilmenite structure has been previously reported by Spiegelberg [3]. A body centered cubic solid solution phase originally reported as KSbO_3_ [3] has been found to occur metastably in the system. The 3K_2_O:5Sb_2_O_5_ compound was originally reported by Aurivillius [4] and this phase, orthorhombic with *a* = 24.274, *b* = 7.157, *c* = 7.334 A, space group Pbam and the new compound K_2_O:2Sb_2_O_5_, monoclinic with *a* = 19.473, *b* = 7.452, *c* = 7.198 A, *β* = 94 °54.4′ space group C2/m were reported by H.Y–P Hong [5].

Two stable polymorphs of Sb_2_O_4_ have been reported in the literature. They are *α–*Sb_2_O_4_ which is orthorhombic [6] *a* = 5.436, *b* = 11.76 and *c* = 4.81 A and *β–*Sb_2_O_4_ which is monoclinic [7] *a* = 11.905, *b* = 4.834, *c* = 5.383 A, *β* = 101°22′. In the following discussion all ratios (1:3, 3:5, etc.) refer to the alkali/metal atom ratio rather than to the particular starting material that may have been used.

## 2. Specimen Preparation and Test Methods

In order to minimize the effect of foreign anion contamination in Sb_2_O_4_, high purity antimony metal was ground and oxidized on Pt setters in air at elevated temperatures. It was found that the formation of a thin antimony oxide coating at 450 °C prevented further surface reaction of antimony with the platinum. Once this coating was formed the temperature could be raised to 500 *°*C for relatively rapid oxidation without reaction with the platinum setter. Spectrographs analysis of Sb_2_O_4_ indicated that platinum contamination was only 2 ppm. The only other metals found in quantities greater than the detectable limit were traces of Zr and Tb.

Mixtures of Sb_2_O_4_ with sodium or potassium carbonate were prepared by weighing to the nearest ±0.1 mg in sufficient quantities to yield a 1 g batch. No corrections were made for percentage purity except loss on ignition. Each batch was hand mixed under acetone with a mortar and pestle. The mixtures were placed on setters fabricated from platinum foil and calcined in air at 500 °C for 60 hs. Following this preliminary calcine the mixtures received a second calcine at 700 °C for 60 hs. In the K_2_O–Sb_2_O_4_ system the specimens received a third calcining at higher temperatures in a small platinum tube for 1 h. To minimize volatility at higher temperatures, sealed platinum tubes approximately 2 cm long were employed for all succeeding experiments unless otherwise stated. About one-third of the volume was occupied by the specimen and the remainder of the tube was flattened prior to sealing. At elevated temperatures the expansion of the flattened portion of the tube provided the necessary additional volume for expansion of the vapors without rupture. The actual pressure inside the tube is unknown. At elevated temperatures the time of the experiment was shortened to minimize “soaking in” of Sb_2_O_4_ into the platinum. By employing this procedure it was felt that the best approach to equilibrium was achieved. Sub-solidus and melting point values were obtained by quenching specimens sealed in platinum tubes and examining them at room temperature.

An electrically heated vertical tube furnace was used for quenching. The temperature was controlled to about ±2 °C. Temperatures were measured with a Pt versus Pt 10 percent Rh thermocouple which was calibrated several times during the course of the work. Due to the marked volatility of the antimonates and the reactivity of the alkaline materials at elevated temperatures, thermocouple contamination sometimes resulted. To minimize this contamination problem the thermocouples were changed frequently. The overall accuracy of the reported temperature is estimated to be about ±5 °C.

The first sign of glazing of the specimen surface established the solidus values. The few liquidus values that are reported were established by the formation of a concave meniscus. No attempt was made to obtain liquidus values in the Sb_2_O_4_-rich portion of these systems because of the high vapor pressure.

Equilibrium is generally considered to have been obtained when x-ray diffraction patterns of specimens successively heated for longer times and/or at higher temperatures show no change. X-ray powder diffraction patterns were made using a high angle recording Geiger counter diffractometer and nickel-filtered copper radiation with a scan rate of 1/4° 2*θ*/min and a chart speed of 1/4 in/min. The unit cell dimensions reported can be considered accurate to about ±5 in the last decimal place listed.

## 3. The System Sb_2_O_4_–NaSbO_3_

**Figure 1:**
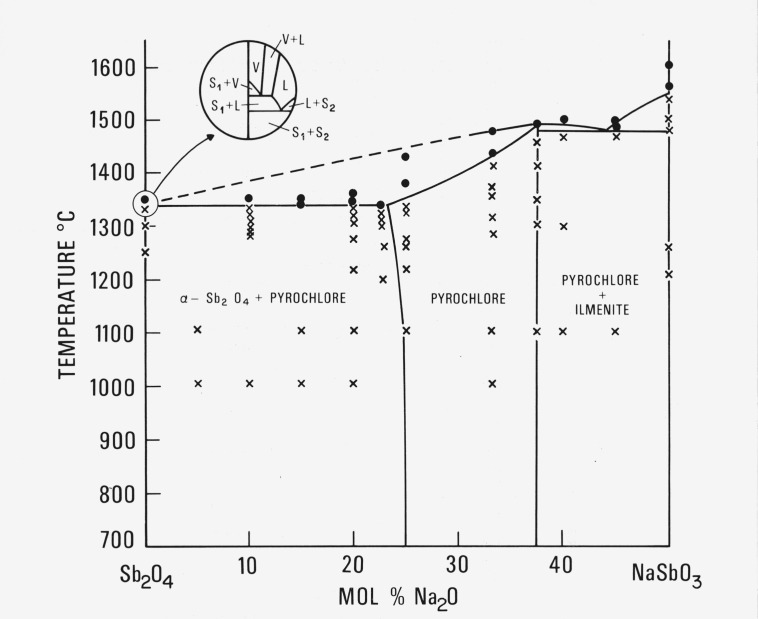
Phase equilibrium diagram for the system *Sb_2_O_4_–NaSbO_3_* Not necessarily a true binary system. L = liquid, S = solid, V = vapor, S_1_ = *α −* Sb_2_O_4_, S_2_ = pyrochlore. ●—melting ×—no melting

The system between the compositional limits of NaSbO_3_ and Sb_2_O_4_ has been examined in detail. The phase equilibrium diagram, figure 1, has been constructed from the data given in table 1. When Sb_2_O_4_ is reacted at low temperature (500–1000 °C) with alkali carbonate it generally loses CO_2_ and gains oxygen from the atmosphere to satisfy the equilibrium conditions of the phases formed, which may involve oxidation of the antimony ions. It is therefore understood that the phase diagrams determined in the antimonate systems reported here may not be strictly binary.

The compound NaSbO_3_ (ilmenite-type) was found in this work to melt at about 1555 ± 5 °C. An intermediate pyrochlore solid solution exists from about 37.5 mol percent Na_2_O:62.5 mol percent Sb_2_O_4_ to 24 mol percent Na_2_O:76 mol percent Sb_2_O_4_ at 1350 °C. The 1:3 composition probably does not really correspond structurally to [NaSb^+3^]Sb_2_^+5^O_7_ although the 3:5 composition may be written as $\left[{\text{Na}}_{1.5}{\text{Sb}}_{0.5}^{+3}\right]{{\text{Sb}}_{2}}^{+5}O_{6.5}$—see section 6.1. The 3Na_2_O: 5Sb_2_O_4_ composition apparently melts congruently at 1490 ± 5 °C. The solidus curve falls from this temperature to about 1340 ± 5 °C at 24 mol percent Na_2_O:76 mol percent Sb_2_O_4_. A two phase region exists between the pyrochlore solid solution and Sb_2_O_4_. An unknown phase was found to occur in the system which could be made approximately single phase by calcining the composition 15 mol percent Na_2_O:85 mol percent Sb_2_O_4_ at 750 °C and reheating in a sealed Pt tube to 1000 °C for 64 h in the presence of PtO_2_. This phase has an as yet unindexed x-ray diffraction pattern with the four strongest lines occurring at *d* values equal to 2.283, 2.798, 3.453, 8.23 A.

In the Sb_2_O_4_ rich portion of the system from 10 percent Na_2_O (or K_2_O):90 percent Sb_2_O_4_ to 100 percent Sb_2_O_4_ experimental interpretation at or near the liquidus is exceedingly difficult since the conventional picture of solid and liquid is not evident. At the composition 15 mol percent Na_2_O (or K_2_O): 85 mol percent Sb_2_O_4_, quenched liquid plus solid is evident. From this data the solidus can be delineated. However at or near Sb_2_O_4_, the solid appears to transform to vapor with no indication of the liquid phase. The most likely interpretation of the data is shown in the circular insert in figure 1, indicating that solid Sb_2_O_4_ + solid pyrochlore_ss_ melts to solid pyrochlore_ss_ and liquid. Within experimental error, the sublimation and eutectic points appear to be at the same temperature and the field Sb_2_O_4_ + Liq (labeled S_1_ + L) is not seen.

### 3.1. NaSbO_3_

The compound NaSbO_3_ was first reported by Schrewelius [1] to be hexagonal, *a* = 5.316 and c = 15.95 A with an ilmenite structure. This compound was found in the present work to melt congruently at about 1555 ± 5°C. No other stable polymorphs were encountered.

### 3.2. Pyrochlore Solid Solution

One intermediate phase, a cubic pyrochlore solid solution was characterized in the system. The compositional range varies from approximately Na_2_O:3Sb_2_O_4_ to 3Na_2_O:5Sb_2_O_4_ with unit cell dimensions varying from 10.289 to 10.286 A respectively. Since the pyrochlore is a tunnel structure and this pyrochlore is the only sodium containing pyrochlore reported that can be formulated by direct synthesis it was worthy of further study as a possible ionic conductor. For ionic conductivity measurements dense materials were needed and several experiments were conducted with Na_2_O:2Sb_2_O_4_ in an effort to determine the stability of the pyrochlore solid solution under high pressure and temperature. Samples in sealed platimun tubes were heated at 1100 °C and 4000–5000 psi 2 for several hours. The resulting specimens are single phase pyrochlore which appear to be very dense. The average density of four measured fragments was 5.26 ± 0.05 g/cm^3^.

For ionic conductivity measurements, pellets of Na_2_O:2Sb_2_O_4_ (1.9 cm in diameter) were placed in sealed platinum foil envelopes and hot pressed by a commercial company at 1100°C and 5,000 psi. The pellets were single phase material with a density of 96 percent theoretical (see sec. 6.1). The ionic conductivity of these pellets was measured at NASA Lewis Research Center [8] and they were found to be essentially insulators.

The distribution of the various ions (i.e., Na^+^, Sb^+3^, Sb^+5^, O^−2^) in the Na_2_O:2Sb_2_O_4_ specimen is currently being determined at NBS from single crystal structure analysis. Until the results of this analysis are forthcoming it may be assumed that the “lone pair” electrons associated with Sb^+3^ will not allow the passage of Na^+^ through the channels.

### 3.3. Polymorphism of Sb_2_O_4_

Two stable polymorphs of Sb_2_O_4_ have been reported in the literature. They are *α*-Sb_2_O_4_, which is orthorhombic [6], *a* = 5.436, *b* = 11.76, *c* = 4.810 A and *β*-Sb_2_O_4_, which is monoclinic [7], *a* = 11.905, *b* = 4.834, *c* = 5.383 A and *β* = 101°22′. From table 2a it can readily be seen that specimens quenched from a temperature-composition region represented on the phase diagram, figure 1, as Sb_2_O_4_+ pyrochlore may contain either *α*-Sb_2_O_4_ and/or *β*-Sb_2_O_4_ when quenched from high temperatures and ambient pressures and examined at room temperature. From this seemingly inconsistent data it would appear that *α*-Sb_2_O_4_ and *β*-Sb_2_O_4_ have a polytypic relationship. To help resolve this problem a high resolution electron microscope study should be done.

From the data in table 2b it appears that the *β* form is the equilibrium high pressure form of Sb_2_O_4_. Insufficient data have been collected to establish if an equilibrium boundary curve exists between *α*-Sb_2_O_4_ and *β*-Sb_2_O_4_ at various temperatures and pressures. When specimens are sealed and heated under pressure in the presence of PtO_2_ in either Pt or Au tubes single phase *β*-Sb_2_O_4_ is obtained. However when heated under pressure without the PtO_2_, a two phase specimen results, *β*-Sb_2_O_4_ and the dense high pressure form of Sb_2_O_3_ (valentinite). A similar polytypic relationship probably exists for the two polymorphs of Sb_2_O_3_.

## 4. The System Sb_2_O_4_–KSbO_3_

**Figure 2:**
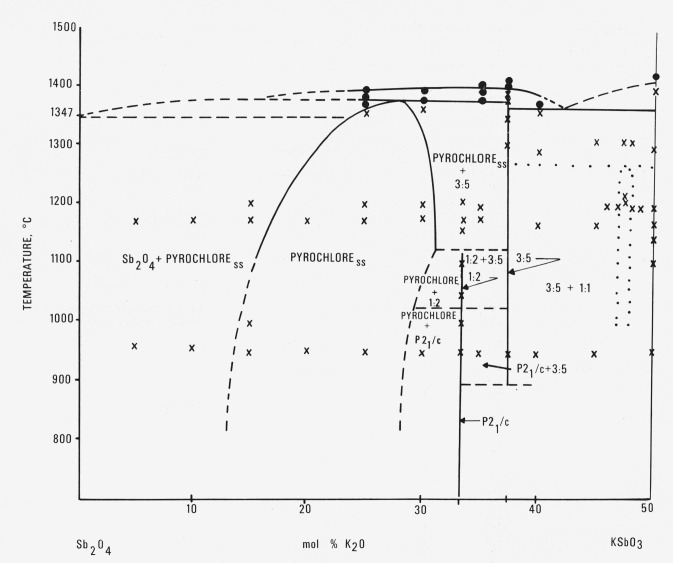
Phase equilibrium diagram for the system *Sb_2_O_4_–KSbO_3_* Not necessarily a true binary system ○—melting ×—no melting ss—solid solution 1:2—K_2_O:2Sb_2_O_5_ 3:5—3K_2_O:5Sb_2_O_5_ P2_1_/c—lower temperature form of K_2_O:2Sb_2_O_5_

This system has been examined between the compositional limits of KSbO_3_ and Sb_2_O_4_. The results are given in the data presented in table 3 from which the phase relationships have been established as shown in figure 2.

### 4.1. Compounds in the System

The compound KSbO_3_ with an ilmenite structure *a* = 5.361, *c* = 18.213, was previously reported [3] and was found in this work to melt congruently at 1420±5°C. A body centered cubic solid solution phase originally reported as KSbO_3_ [3] has been found to occur metastably at about 47.5 percent K_2_O. The 3K_2_O:5Sb_2_O_5_ compound was found to melt congruently at about 1450 °C. The K_2_O:2Sb_2_O_1_ compound was found to have a phase transition at about 1000 °C and to dissociate to pyrochlore plus 3K_2_O:5Sb_2_O_5_ at about 1150 °C. The low temperature form of K_2_O:2Sb_2_O_5_, labeled P2_1_/*c*, represents a monoclinic phase with *a* = 7.178, *b* = 13.378, *c* = 11.985 A and *β* = 124°10′. Single crystals of this phase were grown by flux evaporation from the composition 50K_2_O:5Sb_2_O_4_:45MoO_3_. The unit cell and space group were determined from these crystals and confirmed by least square indexing of the powder diffraction pattern of the low temperature form of the compound K_2_O:2Sb_2_O_5_. The pyrochlore solid solution exists at 1150°C from about 15 mol percent K_2_O:85 mol percent Sb_2_O_4_ to greater than 30 mol percent K_2_O:70 mol percent Sb_2_O_4_. The melting characteristics of these phases have been partially determined as shown in table 3 and figure 2.

### 4.2 Hydroxyl Ion Stabilization of Cubic Potassium Antimonate

The compound KSbO_3_ was reported previously as being cubic at ambient conditions after treatment at high temperatures and pressures [9].

In the current work, occasional small amounts of a cubic phase were seen in the x-ray powder diffraction pattern of KSbO_3_ ilmenite heated at ambient pressure. For these reasons, specimens of 1:1 and 3:5 mol ratios K_2_O:Sb_2_O_4_ were equilibrated in air at 750 °C for 60 h to oxidize and form the phases KSbO_3_ and K_3_Sb_5_O_14_ and then reheated for 1 h at 1200°C to drive off all excess moisture. X-ray diffraction patterns of these specimens showed single phase ilmentite and the 3K_2_O:5Sb_2_O_5_ compound. Portions of these 1200°C calcines were then weighed and mixed in acetone in the appropriate ratios to yield compositions of 46, 47, 47.5, 48 and 49 mol percent K_2_O. Each of these specimens was dried at 240°C for 1 h and heated in open Pt tubes at 1200°C for 1 h. Only the x-ray pattern of the 46 percent specimen showed a small amount of 3K_2_O:5Sb_2_O_5_, the others contained only the cubic phase. A new specimen of of 48 mol percent K_2_O was prepared in the same way except the Pt tube was sealed. After 1 h at 1200 °C, the x-ray pattern of the specimen showed only about 50 percent cubic. A new specimen of 48 percent K_2_O was prepared by weighing the 1:1 and 3:5 phases immediately after removal from the 1200 °C furnace and sealing the material in a flattened Pt tube within 1–2 min. This tube was then inflated at 1200 °C for a few minutes and the material mixed by shaking in a “wiggle-bug.” The sealed specimen was then heated for 64 h at 1200 °C. The resultant specimen had exceedingly large grain growth indicating considerable solid state recrystallization but showed *no* cubic phase. The conclusion is inescapable that access to atmospheric moisture is probably *necessary* for the formation of the cubic phase at atmospheric pressure.

A paper entitled “Flux Synthesis of Cubic Antimonates” was published by the present authors during the course of this work [10]. In addition to the discovery that the F^−^ ion stabilized the formation of the body centered cubic phase of potassium antimonate it was disclosed that the cubic antimonate could also be obtained by reacting KSbO_3_ with a small amount of other cations with small radii like B^+3^, Si^+4^, etc. It now appears obvious that in this reaction the boron or silicon (etc.) actually ties up some of the K^+^ ion in a second phase and allows the K^+^ deficient antimonate to react with atmospheric moisture to form the cubic antimonate previously thought to be “KSbO_3_.”

## 5. The Systems of NaSbO_3_ With Additions

### 5.1. The System NaSbO_3_-NaF

To determine if NaF additions will stabilize the body-centered cubic phase, similar to the 6KSbO_3_: KF-phase [10], NaF was added to NaSbO_3_ in the ratio of 3NaSbO_3_:NaF, 4NaSbO_3_:NaF, 5NaSbO_3_: NaF and 6NaSbO_3_:NaF. After heating at 750 °C and 1000 °C in sealed Pt tubes, the x-ray patterns showed only ilmenite and NaF, however after heating at ~1150 °C all the compositions contained some body centered cubic-type phase. The compositions 3NaSbO_3_:NaF and 4NaSbO_3_:NaF, when heated in sealed Pt tubes at ~1250 °C, did not contain ilmenite and appeared to be the closest to single phase cubic. The small crystals of 4NaSbO_3_:NaF prepared by quenching in a small sealed tube appeared to be well-formed truncated octahedrons. However, the room temperature x-ray diffraction pattern of the material had somewhat diffuse lines, with the exception of the *h*00 lines which were reasonably sharp, suggesting rhombohedral symmetry. This material was placed on a hot stage microscope slide and analyzed by x-ray diffraction from room temperature up to 220 °C. At 190 °C the material appeared to start to go cubic and by 220 °C a good quality cubic x-ray diffraction pattern was obtained (*a* = 9.353 A). When the material was cooled to room temperature the symmetry was again non-cubic. As the *h*00 lines deteriorate somewhat on cooling, the true symmetry of the room temperature form is probably no higher than monoclinic or triclinic rather than rhombohedral. It was therefore not unreasonable to expect that a body centered cubic phase could be obtained by direct synthesis with NaF without the necessity for Na^+^ ion exchange.

### 5.2. The Ternary System NaSbO_3_:Sb_2_O_4_:NaF

**Figure 3:**
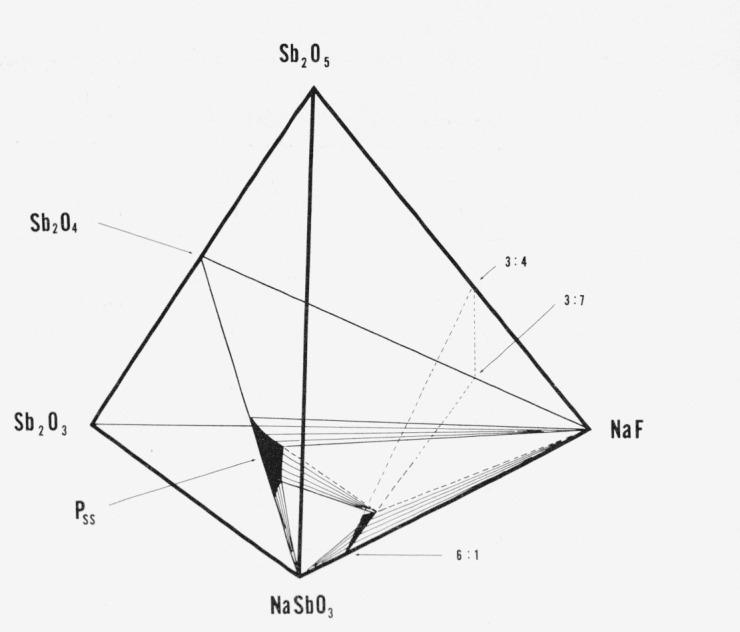
Phase relations in the quaternary system *NaSbO_3_–Sb_2_O_3_–Sb_2_O_5_–NaF*. The join 6:1—3:4 represents the formula
$\left[{□}_{2+x}{\text{Na}}_{14-x}F_{2}\right]\left[{{\text{Sb}}_{12}}^{+5}O_{36-x}F_{x}\right].$ The join 6:1—3:7 represents the formula
$$\left[{□}_{2}{\text{Na}}_{14}F_{2}\right]\left[{{\text{Sb}}_{y}}^{+3}{\text{Sb}}_{12-y}^{+5}O_{36-\left(x+2y\right)}F_{\left(x+2y\right)}\right]$$

**Figure 4:**
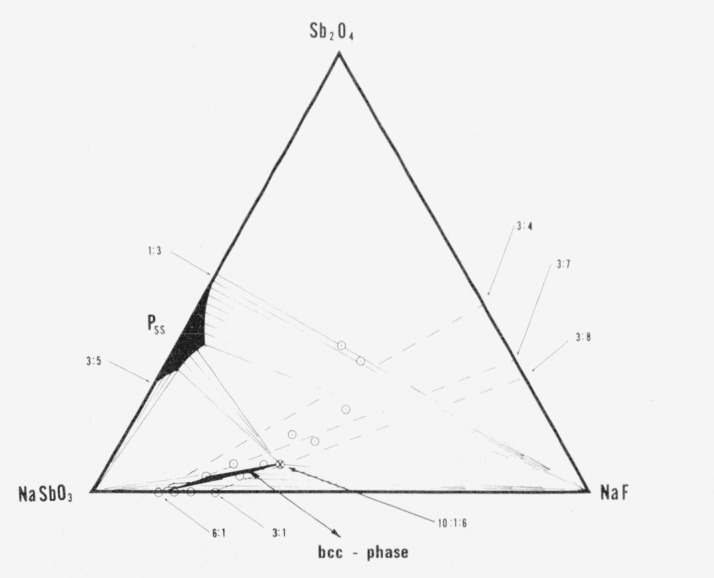
Phase relations in the ternary system *NaSbO_3_–Sb_2_O_4_–NaF*. The join 6:1—3:4 represents the formula
$\left[{□}_{2+x}{\text{Na}}_{14-x}F_{2}\right]\left[{{\text{Sb}}_{12}}^{+5}O_{36-x}F_{x}\right]$ The join 6:1—3:7 represents the formula
$\left[{□}_{2}{\text{Na}}_{14}F_{2}\right]\left[{{\text{Sb}}_{y}}^{+3}{{\text{Sb}}_{12}}^{+5}O_{36-\left(x+2y\right)}F_{\left(x+2y\right)}\right]$ The join 3:1—3:8 represents the formula
$$\left[{\text{Na}}_{16}F_{2}\right]\left[{\text{Sb}}_{2+x}^{+3}{\text{Sb}}_{10-x}^{+5}O_{34-2x}F_{2+2x}\right]+O_{2}.$$

X-ray diffraction patterns (single crystal and powder) of selected NaF-flux synthesized [11] washed crystals show only a truly cubic body centered phase (*a* = 9.334 A). It must be postulated that the composition formed by this technique is slightly different from that made essentially single phase at 4NaSbO_3_:NaF in a sealed tube. In an attempt to obtain a fluorine-substituted body centered cubic phase which exists at room temperature the compositions shown in table 4 were prepared and show the reported phases when quenched from 1250 °C. Equilibrium was not obtained in overnight heat treatments at 1200 °C. At 1350 °C the body centered cubic phase started to decompose. The composition 68NaSbO_3_:4Sb_2_O_4_:28NaF (mol %) was chosen as the best composition for further studies on ceramic procedures [11]. The phases found in the specimens heated at ~1250 °C are summarized in “equilibrium” diagrams for the quaternary system NaSbO_3_–Sb_2_O_3_–Sb_2_O_5_–NaF (fig. 3) and the ternary plane of this system NaSbO_3_–Sb_2_O_4_–NaF (fig. 4).

## 6. Relation of Structural Mechanisms of Non-Stoichiometry to Ionic Conductivity

It is probably generally accepted that a phase which exhibits unusual ionic conductivity must necessarily be structurally non-stoichiometric. Unfortunately the opposite is not necessarily true. Nevertheless a crystallographic understanding of non-stoichiometric phases is an obvious necessity to the tailoring of new alkali ion conductors. For this reason it is worthwhile to discuss the nature of the non-stoichiometry which has been observed in this study for those phases which seem to be of interest.

### 6.1. Pyrochlore Phases

In the KTaO_3_–WO_3_ system a pyrochlore phase occurs at about the 1:1 ratio or K_1.0_[TaW]O_6_ [11, 12]. Unfortunately, the pyrochlore in this system transforms to a tetragonal tungsten bronze (TTB) at high termperatures. Although it can be ion exchanged with Na^+^ to produce an ion conducting pyrochlore phase, this phase is not stable above about 450 °C [11]. The only stable Na^+^ containing pyrochlore is the one in the Sb_2_O_4_–NaSbO_4_ system and apparently this one is not a good ionic conductor.

The distribution of Na^+^, Sb^+3^, Sb^+5^ and O^−2^ ions in a pyrochlore single crystal is currently under evaluation by the Crystallography Section at NBS. However, certain assumptions can be made which may enable us to postulate the approximate distribution. The formula for the compositions observed to result in a pyrochlore structure night be postulated to be [NaSb^+3^]Sb_2_O_7_ for the Na/Sb ratio of 1:3 $\left[{\text{Na}}_{1.33}{\text{Sb}}_{0.67}^{+3}\right]{\text{Sb}}_{2}O_{6.67}$ for 1:2, and [Na_1.5_Sb_0.5_]Sb_2_O_6.5_ for 3:5. However, these compositions do not illustrate the structural nature of pyrochlore nor account for the observation that the “lone pair” electrons associated with Sb^+3^ will not allow O^−2^ ions to completely coordinate the antimony and result in apparent vacancies.

The structural formula of pyrochlore should be written as [A_2_X][B_2_X_6_] to emphasize the fact that the octahedral network of B_2_X_6_ is required to be complete if the structure is to be stable. The A_2_X ions fill the intersecting channels in this B_2_X_6_ framework. In our material the B_2_X_6_ framework must be represented as [Sb_2_^+5^O_6_]^−2^ and *must* be stoichiometric. All remaining Na^+^ and O^−2^ ions, as well as Sb^+3^, must be in the [A_2_X]^+2^ portion of the formula. All Sb^+5^ must be in B_2_X_6_ and only Sb^+3^ in A_2_X. Furthermore the *maximum* number of the sum of Na^+1^, Sb^+3^, excess O^−2^ (beyond O_6_^−2^) *and* “lone pair” electrons cannot exceed three. One can then write the general formula as [A_2_O]^+2^[Sb_2_O_6_]^−2^ with [A_2_O]^+2^ equal to

$$\left[{\text{Na}}_{2/k}^{+1}+{{\text{Na}}_{x}}^{+1}+{{\text{Sb}}_{kx}}^{+3}+{O_{y}}^{-2}+L.P._{kx}\right]\leq 3$$

where *k* equals the ratio Sb/Na. Using the ionic valences and the sum of the ions equal to three, *maximum* densities can be calculated and compared with the observed to test the structural hypothesis. The *maximum* density for the Na/Sb ratio of 1:3 represented by the formula

$${\left[{\text{Na}}_{0.917}^{+1}{\text{Sb}}_{0.75}^{+3}O_{0.583}^{-2}{□}_{0.75}\right]}^{+2}{\left[{{\text{Sb}}_{2}}^{+5}O_{6}\right]}^{-2}$$

is calculated to be 5.469 g/cm^3^.

For the Na/Sb ratio of 3:5 with the formula

$${\left[{{\text{Na}}^{+1}}_{1.5}{{\text{Sb}}^{+3}}_{0.5}{O^{-2}}_{0.5}{□}_{0.5}\right]}^{+2}{\left[{\text{Sb}}_{2}O_{6}\right]}^{-2}$$

the density is calculated as 5.406 g/cm^3^. For the intermediate composition with the Na/Sb ratio of 1:2 and a formula of ${\left[{\text{Na}}_{1.294}^{+1}{\text{Sb}}_{0.588}^{+3}O_{0.529}^{-2}{□}_{0.588}\right]}^{+2}{\left[{\text{Sb}}_{2}O_{6}\right]}^{-2}$ the *maximum* density is found to be 5.481 g/cm^3^. The density found for our isostatically hot pressed specimens is 96.0 percent of the *maximum theoretical* density. It should be remembered however that the true theoretical density of any given Sb/Na ratio will decrease with decrease in temperature. Thus the densities obtained on our hot pressed specimens are, in all probability, greater than 96 percent of theoretical in view of the expected increased oxidation of the Sb at the relatively low temperatures involved.

### 6.2. Body Centered Cubic Antimonates

A successful method of synthesizing cubic potassium antimonate by heating in molten KF was published by the present authors [10]. The major reason for the success in obtaining completely single phase fluorine stabilized cubic potassium antimonate is that the KSbO_3_ ilmenite form is H_2_O soluble and may be easily separated from the cubic material.

An examination of the structural model of the octahedral framework of the body centered cubic antimonate phase suggests that this structure *must* always have some anion (X) occupancy in the 000 and 1/2 1/2 1/2 positions. The structural formula thus appears to be [A_16_X_2_]^+12^[Sb_12_O_36_]^−12^ with the alkali ion in position (A) located at (or just off) the juncture of the open cages. However, it seems very likely from both structural reasons (bond lengths, etc.) and valency considerations that either or both of the nonframework positions will be non-stoichiometric. Valency considerations require that at least two out of 16 alkali ions must be missing and the structural formula then becomes

$${\left[{□}_{2}A_{14}X_{2}\right]}^{+12}{\left[{\text{Sb}}_{12}O_{36}\right]}^{-12}$$

This formula corresponds to the composition reported by Goodenough, et al. [13] for the single crystal x-ray diffraction analyses of the phase synthesized with KF according to the NBS method [10]:

$$K_{12}{\text{Sb}}_{12}O_{36}.2\text{KF or}\;{\left[{□}_{2}K_{14}F_{2}\right]}^{+12}{\left[{\text{Sb}}_{12}O_{36}\right]}^{-12}.$$

It seems quite likely, however, that this general formula does not completely account for all of the preparations which have been observed to form this structure, whether body centered or primitive. The observation that a primitive phase can be formed, in air, by reaction with atmospheric moisture at a 48:52 ratio suggests that this phase may well have considerably less than 14 alkali ions per unit cell. The formula *must* be compensated, in this case, by a substitution of a monovalent anion [(OH)^−^, F^−^] in the *octahedral framework.* The general formula then becomes [□_2+_*_x_*A_14−_*_x_*X_2_]^+(12−^*^x^*^)^ [Sb_12_O_36−_*_x_*,X*_x_*]^−(12−^*^x^*^)^. The composition found at ~48:52 in the potassium antimonate system can be written (assuming a ratio of 11:12 K/Sb or 47.826% K_2_O):

$$K_{22}{\text{Sb}}_{24}O_{71}+5H_{2}O\to K_{22}{\text{Sb}}_{24}O_{66}{\left(\text{OH}\right)}_{10}$$

or

$${\left[{□}_{5}K_{11}{\left(\text{OH}\right)}_{2}\right]}^{+9}{\left[{\text{Sb}}_{12}O_{33}{\left(\text{OH}\right)}_{3}\right]}^{-9}$$

which also can be described as 6KSbO_3_:3Sb_2_O_5_:5KOH The general formula describing the K^+^ containing compositions is then

$${\left[{□}_{2+x}K_{14-x}X_{2}^{\prime }\right]}^{+\left(12-x\right)}{\left[{{\text{Sb}}_{12}}^{+5}O_{36-x}X_{x}^{\prime }\right]}^{-\left(12-x\right)}.$$

The above formula contains only pentavalent antimony and apparently does not completely explain the compositions which form a “stable” body centered cubic phase in the system NaSbO_3_: Sb_2_O_4+_*_x_*:NaF. The only formula which does not involve the loss or gain of O^−2^ (or F^−^) when the Sb_2_O_4_ is added in a sealed tube corresponds to:

$$\left[{□}_{2}{\text{Na}}_{14}F_{2}\right]\left[{{\text{Sb}}_{y}}^{+3}{\text{Sb}}_{12-y}^{+5}O_{36-2y}F_{2y}\right]$$

which is represented by the join 6:1—3:7 on figures 3 and 4. There is really no place in the framework structure for Sb^+3^ and it is difficult to believe that octahedrally coordinated antimony can be Sb^+3^. However, for convenience, the formulas can be written involving Sb^+3^. The new formula would then have two variables:

$$\left[{□}_{2+x}A_{14-x}X_{2}^{\prime }\right]\left[{{\text{Sb}}_{y}}^{+3}{\text{Sb}}_{12-y}^{+5}O_{36-\left(x+2y\right)}X_{\left(x+2y\right)}^{\prime }\right]$$

represented by the plane in the quaternary system NaSbO_3_:Sb_2_O_3_:Sb_2_O_5_:NaF bounded by the 6:1—3:4 and 6:1—3:7 joins of figures 3 and 4. However the single phase region in this system actually appears to contain more NaF than described by this general formula. Apparently some O_2_ is evolved in the sealed Pt tubes, the amount depending on uncontrolled variables such as the amount of free volume in the tube and on changes from the original composition during treatment. The absolute maximum amount of NaF which can be accommodated structurally by the body centered cubic phase can be described by the formula

$${\left[{\text{Na}}_{16}F_{2}\right]}^{+14}{\left[{\text{Sb}}_{2-x}^{+3}{\text{Sb}}_{10-x}^{+5}O_{34-2x}F_{2+2x}\right]}^{-14}$$

which represents a line in the system shown by the join 3:1—3:8 in figure 4 and involves the evolution of one molecule of gas (O_2_) per formula unit. The results of our investigations so far suggest that the body centered phase approaches this formula as a limit. The composition of the cubic phase in equilibrium with excess Sb_2_O_4_ and molten NaF actually appears to touch this line at approximately 10NaSbO_3_:Sb_2_O_4_:6NaF or

$$\left[{\text{Na}}_{16}F_{2}\right]\left[{{\text{Sb}}_{3}}^{+3}{{\text{Sb}}_{9}}^{+5}O_{32}F_{4}\right]+O_{2}.$$

The single phase distorted cubic material on the binary join NaSbO_3_:NaF appears to have a composition between 6:1 and 5:1 or approximately 11NaSbO_3_:2NaF or

$$\left[{\text{Na}}_{14.18}F_{2}\right]\left[{\text{Sb}}_{12}O_{35.818}F_{0.1818}\right]+0.0909\;O_{2}.$$

The compositions in the quaternary system thus probably lie on a join between these two end members.

## Figures and Tables

**Table 1:** Experimental data for compositions in the system sodium antimonate-antimony tetroxide

Composition		Heat Treatment^a/^		Results		
Na_2_O mol%	Sb_2_O_4_ mol%	Temp °C	Time hr	Physical Observation		X-ray Diffraction Analysis^b/^
50	50	1213		not melted		
		1264	3	″	″	NaSbO_3_
		1435	1	″	″	″
		1484	1	″	″	
		1502	.08	″	″	
		1542	.08	″	″	NaSbO_3_ + unknown
		1569	.08	melted		
		1602	.08	″		
45	55	1000	48	not melted		
		1100	48	″	″	NaSbO_3_ + pyrochlore ss
		1473	.25	″	″	
		1488	.08	partially melted		
		1495	.08	completely melted		
40	60	1102	20	not melted		pyrochlore ss + NaSbO_3_
		1305	19	″	″	″ ″
		1430	.08	″	″	
		1470	.08	″	″	
		1488	.08	″	″	
		1495	.03	completely melted		
37.5	62.5 (3:5)	1100	48	not melted		Pyrochlore ss
		1192	1	″	″	
		1306	19	″	″	pyrochlore ss
		1326	20	″	″	
		1351	1	″	″	
		1373	2	not melted (reheat of 1100–4)		pyrochlore ss
		1391	2	not melted		
		1392	.16	″	″	pyrochlore ss
		1412	.16	″	″	″
		1447	.16	″	″	
		1454	.33	″	″	
		1458	.08	″	″	
		1464	.08	″	″	
		1476	.08	″	″	
		1487	.08	″	″	
		1490	.08	melted		
33.33	66.67 (1:2)	1000^c/^	8	not melted		
		1009^d/^	168	″	″	pyrochlore ss
		1100	3	″	″	″
		1103^e/^	91	″	″	″
		1287	2	″	″	″
		1292	1.5	″	″	″
		1306	24	″	″	
		1307	19	″	″	pyrochlore ss
		1316	.5	″	″	″
		1317	3.5	″	″	
		1354	.75	″	″	pyrochlore ss
		1360	24	″	″	″
		1376	.5	″	″	″
		1378	.5	″	″	
		1411	19	″	″	
		1418	.02	″	″	
		1437	24	partially melted		
		1475	.02	completely melted		
25	75 (1:3)	750	60	not melted		pyrochlore ss + unknown^f/^
		800	60	″	″	pyrochlore ss + unknown^g/^
		800	60	″	″	pyrochlore ss + unknown^f/^
		800	336	″	″	pyrochlore ss + unknown
		1098	16	″	″	pyrochlore ss
		1192	1	″	″	″
		1200	24	″	″	″
		1220	2	″	″	″
		1277	2	″	″	
		1306	24	″	″	
		1307	.08	″	″	
		1317	16^h/^	″	″	pyrochlore ss
		1325	1	″	″	
		1339	.08	″	″	
		1345	.25	″	″	pyrochlore ss
		1346	.08	″	″	
		1358	.08	″	″	
		1377	.02	partially melted		pyrochlore ss + NaSbO_3_
		1427	.02	″	″	
23	77	1200	24	not melted		pyrochlore ss + β–Sb_2_O_4_
		1266	4	″	″	pyrochlore + α + β^i/^
		1267	19	″	″	pyrochlore ss^i/^
		1299	.08	″	″	
		1304	.08	″	″	
		1313	.08	″	″	
		1322	.08	″	″	
		1332	.08	″	″	pyrochlore ss^i/^
		1338	.08	″	″	
20	80	1099	672	not melted		pyrochlore ss^k/^
		1107	144					
		1200	24	not melted		pyrochlore ss + α
		1220	2.5	″	″	pyrochlore ss + α-Sb_2_O_4_
		1234	2.5	″	″	″ ″
		1277	16	″	″	pyrochlore ss + β-Sb_2_O_4_
		1301	.5	not melted		—
		1305	19	″	″	NaSbO_3_^k/^
		1306	24	″	″	pyrochlore ss + β-Sb_2_O_4_
		1314	.08	″	″	pyrochlore ss + α-Sb_2_O_4_
		1318	.08	″	″	
		1335	.08	″	″	
		1339	.2	″	″	
		1340	.08	″	″	
		1345	.2	partially melted		
		1362	.5	″	″	
15	85	800	74	not melted		unknown + pyrochlore ss + α-Sb_2_O_4_^g^,^l/^
		800	60	″	″	α + pyrochlore ss + unknown^l/^
		1000	64			unknown + tr α-Sb_2_O_4_ (dried 240)^k^,^l/^
		1000	64			unknown + tr α-Sb_2_O_4_^k^,^l/^
		1007	48	not melted		pyrochlore + α-Sb_2_O_4_ + unknown^l/^
		1107	144	″	″	pyrochlore ss + a-Sb_2_O_4_ + β-Sb_2_O_4_
		1200	24	″	″	
		1200	60	″	″	pyrochlore ss + α-Sb_2_O_4_
		1337	.2	″	″	
		1340	.2	″	″	
		1348	.2	partially melted		
10	90	800	74	not melted		α-Sb_2_O_4_ + unknown^l/^
		1007	48	″	″	α-Sb_2_O_4_ + β-Sb_2_O_4_ + pyrochlore ss^l/^
		1107	144	″	″	″ ″ ″
		1234	2	″	″	α-Sb_2_O_4_ + pyrochlore ss
		1281	.33	″	″	″ ″
		1290	.33	″	″	
		1300	.33	″	″	α-Sb_2_O_4_ + pyrochlore ss
		1311	.2	″	″	
		1319	.33	″	″	
		1334	.33	″	″	
		1337	.2	″	″	α-Sb_2_O_4_ + pyrochlore ss
		1351	1	partially melted		α + pyrochlore ss + quenched liquid^m/^
5	95	1007	48	not melted		β-Sb_2_O_4_ + α-Sb_2_O_4_ + pyrochlore ss^i/^
		1107	144	″	″	α-Sb_2_O_4_ + pyrochlore ss + trace β-Sb_2_O_4_^i/^
						β-Sb_2_O_4_^l/^
		1234	3.5	″	″	″ ″ ″ ″

a/ All specimens were preheated to 750°C for 60 hours and 1200°C for 19 hours unless otherwise footnoted. Rate of heating and cooling was approximately 3°/min. For higher heat treatments, speciments were heated in sealed Pt tubes and quenched from temperatures indicated.

b/ The phases identified are given in the order of the amount present (greatest amount first) at room temperature. These phases are not necessarily those present at the temperature to which the specimen was heated.

c/ Specimen heated with PtO_2_ at 68,900 psi in sealed Pt tube.

d/ Specimen heated in sealed Pt tube at 5,000 psi.

e/ Specimen previously heated at 1292°C for 1.5 hours.

f/ Specimen heated in sealed Pt tube in presence of water. The unknown phase formed is probably a hydrate.

g/ Specimen heated in sealed Pt tube in PtO_2_.

h/ Specimen heated in presence of 5:95 Na_2_O:Sb_2_O_4_ which served as a buffer.

i/ In spite of extensive x-ray study it has not been determined which of the polymorphic forms of Sb_2_O_4_ is the stable form.

j/ Sb_2_O_4_ probably soaked into Pt container and the composition changed to pyrochlore ss.

k/ Platinum tube leaked.

l/ Unknown phase, d-spacing of major lines given in text. This phase is probably a hydrated phase which exists in the presence of moisture and/or PtO_2_ and can be eliminated by an additional calcining of 1200°C for several hours. Once eliminated this phase does not appear to reform at lower temperatures in laboratory time.

m/ Specimen contained non-equilibrium material derived from a liquid when quenched from above the liquidus and examined at room temperature.

**Table 2a:** Experimental data for polymorphism in antimony tetroxide

Composition	Heat Treatment		Environment			Results		
Starting Material	Temp °C	Time hr				Physical Observation		X-ray Diffraction Analysis^a/^
α-Sb_2_O_4_	1223	.5	sealed Pt tube			not melted		α + tr β
″	″	.″	unsealed Pt tube			″	″	α
β-Sb_2_O_4_	1223	.5	sealed Pt tube			not melted		β + tr α
″	″	″	unsealed Pt tube			volatilized		–
β-Sb_2_O_4_	1223	2	sealed Pt tube			not melted		β + tr α
α-Sb_2_O_4_	″	″	sealed Pt tube			″	″	α + Sb_2_O_3_
α-Sb_2_O_4_	1303	19	sealed Pt tube			not melted		β + α
β-Sb_2_O_4_	″	″	″	″	″	″	″	β
α-Sb_2_O_4_	1327	.08	sealed Pt tube			not melted		α + β
β-Sb_2_O_4_	″	″	″	″	″	″	″	β + α
α-Sb_2_O_4_	1330	.25	sealed Pt tube			not melted		β + α
α-Sb_2_O_4_	1339	.08	sealed Pt tube			not melted		α + β
β-Sb_2_O_4_	″	″	″	″	″	″	″	β + α
β-Sb_2_O_4_	1345	.08	sealed Pt tube			not melted		β + α
α-Sb_2_O_4_	1350	.08	sealed Pt tube			melted (vapor soaked into Pt)		–
β-Sb_2_O_4_	1350	.08	sealed Pt tube			melted? large tabular vapor grown crystals		–
α-Sb_2_O_4_^b/^	1200	–	high temperature x-ray					α (starting material remained a up to 1200°C)
α-Sb_2_O_4_^c/^	750	24	open tray					α
″	800	″	″	″				″
″	900	″	″	″				α + β
″	950	″	″	″				″ ″

a/ The phases identified are given in the order of the amount present (greatest amount first) at room temperature. These phases are not necessarily those present at the temperature to which the specimen was heated. α refers to α-Sb_2_O_4_ polymorph and β to the β-Sb_2_O_4_ polymorph.

b/ Material placed on platinum slide and heated and examined by x-ray diffraction at various temperatures.

c/ Poorly crystalline as received Sb_2_O_4_ was heated 750°C - 24 hours and the same specimen which was never ground was reheated at 800°C - 24 hours, then 900°C - 64 hours and finally 950°C - 24 hours.

**Table 2b:** Experimental high pressure data for polymorphism in anlimony-tetroxide

Composition Starting Material	Heat Treatment		Environment			Pressure psi	Results^b/^ X-ray Diffraction Analysis
	Temp °C	Time hrs					
α-Sb_2_O_4_^a/^	700	24	Sealed Au tube			88,000	β^c/^ + Sb_2_O_3_^d/^
″	750	48	″	″	″	59,680	″ ″ ″
″	750	96	″	″	″	73,200	″ ″ ″
″	750	16	″	″	″	89,400	β + trace Sb_2_O_3_
″	751	116	″	″	″	109,000	β + Sb_2_O_3_
″	760	96	Sealed Au tube with PtO_2_			80,000	β
″	766	96	Sealed Au tube			88,000	β + Sb_2_O_3_
″	775	115	″	Pt	″	47,500	α + Sb_2_O_3_
″	775	48	″	Pt	″	54,760	β + Sb_2_O_3_
″	775	48	″	Pt	″	66,500	″ ″ ″
″	800	24	″	Au	″	93,000	″ ″ ″
″	800	24	Sealed Au tube with PtO_2_			105,000	β
″	850	16	Sealed Au tube			82,500	β + Sb_2_O_3_
″	900	72	Sealed Pt tube with PtO_2_			104,000	β
β + Sb_2_O_4_	900	72	″ ″ ″ ″ ″			104,000	β

a/ α–Sb_2_O_4_ prepared by the oxidation of Sb at 530°C on Pt tray. This material was reheated at 800°C - 60 hr.

b/ The phases identified are given in the order of the amount present (greatest amount first) at room temperature. These phases are not necessarily those present at the temperatures to which the specimen was heated.

c/ β form of Sb_2_O_4_.

d/ High pressure form of Sb_2_O_3_ (valentinite).

**Table 3:** Experimental data for compositions in the system potassium antimonaie antimony tetroxide

Composition		Heat Treatment^a/^		Results		
K_2_O Mol %	Sb_2_O_4_ Mol %	Temp °C	Time hr	Physical Observation		X-ray Diffraction Analysis^b/^
5	95	950	60	not melted		pyrochlore ss + α-Sb_2_O_4_ + β-Sb_2_O_4_
		1168	48	″	″	α-Sb_2_O_4_ + β-Sb_2_O_4_ + pyrochlore ss^e/^
10	90	950	60	not melted		pyrochlore ss + α-Sb_2_O_4_ + β-Sb_2_O_4_^c/^
		1168	48	″	″	″ ″ ″
15	85	853	24	not melted		
		950	60	″	″	pyrochlore ss
		966	4	″	″	
		1168	48	″	″	pyrochlore ss + α-Sb_2_O_4_
		1200	19	″	″	pyrochlore ss + α-Sb_2_O_4_ + β-Sb_2_O_4_
20	80	950	60	not melted		pyrochlore ss
		1168	48	″	″	″
25	75	950	60	not melted		P2_1_/c^d/^ + pyrochlore ss
		1179	48	″	″	pyrochlore ss
		1361	.08	″	″	
		1375	.08	partially melted		pyrochlore ss
		1385	.08	″	″	
		1403	.08	completely melted		
30	70	950	60	not melted		P2_1_/c^d/^ + pyrochlore ss
		1178	48	″	″	1:2 + pyrochlore ss
		1366	.08	″	″	
		1380	.08	partially melted		pyrochlore ss + 3:5
		1382	.08	″	″	
		1399	.08	completely melted		
33.33	66.67	950	60	not melted		3:5 + P2_1_/c^d/^
		950	64	″	″	
		998	70	″	″	P2_1_/c^d/^
		1050	168	″	″	
		1050^e/^	168	″	″	
		1102	1	″	″	1:2^c/^ + 3:5 + pyrochlore ss + P2_1_/c
		1106	64	″	″	1:2^c/^ + 3:5 + pyrochlore
		1106^f/^	64	″	″	1:2 + 3:5
		1160^f/^	1	″	″	3:5 + pyrochlore ss
		1179	48	″	″	1:2 + 3:5 + pyrochlore ss
		1214^f/^	1	″	″	3:5 + pyrochlore ss
		1214	2	″	″	″ ″
35	65	950	60	not melted		pyrochlore + 3:5 + pyrochlore ss
		1178	48	″	″	1:2 + 3:5
		1380	.08	partially melted		3:5 + pyrochlore
		1397	.08	″	″	
		1409	.08	completely melted		
37.5	62.5	950	60	not melted		
		1174	88	″	″	
		1195	19	″	″	3:5
		1208	1	″	″	3:5 + trace cubic
		950^e/^	64	″	″	
		1310	45	″	″	3:5 + trace 1:1^g/^
		1352	.08	″	″	
		1379	.08	″	″	
		1399	.08	completely melted		
		1416	.08	″	″	
40	60	950	60	not melted		
		1174	88	″	″	3:5 + cubic
		1208	1	″	″	″ ″
		1295^e/^	20	″	″	3:5 + 1:1
		1362^e/^	.5	″	″	″ ″
		1375^e/^	.08	partially melted		
45	55	950	60	not melted		1:1 + cubic + P2_1_/c
		1174	88	″	″	cubic +3:5
		1208	1	″	″	3:5 + cubic
		1311^e/^	1	″	″	3:5 + 1:1
46	54	1200^h/^	1	not melted		cubic +3:5
47	53	1194^h/^	3	not melted		cubic + trace 3:5
		1200	1	″	″	cubic
47.5	52.5	1212^h/^	88	not melted		cubic + 3:5 + 1:1
		1218^h/^	17	″	″	cubic + 1:1 + 3:5
		1310^g^,^h/^	45	″	″	1:1
48	52	1198	3	not melted		cubic
		1200	1	″	″	″
		1200^i/^	1;5	″	″	cubic +3:5 ilmenite
		1308	.5	″	″	1:1
		1103^c/^	1	″	″	cubic + ilmenite + pyrochlore
		1103^i/^	3	″	″	ilmenite + pyrochlore
49	51	1200	1	not melted		cubic
50	50	750	70	not melted		
		800	24	″	″	
		921	1	″	″	
		946	21	″	″	ilmenite
		950	60	″	″	″
		1103	1	″	″	″
		1104	22	″	″	″
		1150	1	″	″	″
		1174	88	″	″	″
		1194	1	″	″	″
		1202	1	″	″	″
		1214	1	″	″	″
		1298	.5	″	″	″
		1363	.5	″	″	″
		1403	.08	″	″	″
		1421	.08	melted		
		1426	.08	″	″	

a/ All specimens were preheated to 500 and 700°C for 60 hours unless otherwise footnoted. Rate of heating and cooling were approximately 3°/min. Specimens were heated in sealed Pt tubes and quenched from temperatures indicated.

b/ The phases identified are given in order of the amount present (greatest amount first) at room temperature. These phases are not necessarily those present at the temperature to which the specimen was heated. 1:2 – K_2_O • 2Sb_2_O_5_; 3:5 – 3K_2_O • 5Sb_2_O_5_ and 1:1 – KSbO_3_ – ilmenite structure.

c/ Non-equilibrium mixture – see Discussion in text.

d/ The phase was indexed from single crystal x-ray precession data which has shown the compound is monoclinic space group P2_1_/c a = 7.178, b = 13.378, c = 11.985, β = 124°10′.

e/ This specimen was previously heated to 500°, 700° and 1200°C – 19 hours in a sealed Pt tube.

f/ Specimen heated in open Pt tube.

g/ Specimen leaked and changed composition.

h/ Composition prepared from a mixture 1:1 and 3:5 – see text for explanation.

i/ Specimen calcined and examined by x-ray diffraction while in form of pellet.

**Table 4:** Experimental data for the ternary system *NaSbO_3_–Sb_2_O_4_–NaF*

Composition	Mol%	Heat Treatment^a/^		X-ray Analysis
		Temp °C	Time hr	
NaSbO_3_	75.08			
Sb_2_O_4_	3.15	1250	19	single phase distorted cubic
NaF	21.77			
NaSbO_3_	67.79			
Sb_2_O_4_	6.25	1250	19	body centered cubic + pyrochlore + ilmenite
NaF	25.96			
NaSbO_3_	53.50			
Sb_2_O_4_	12.34	1250	19	body centered cubic + pyrochlore + sodium fluoride
NaF	34.16			
NaSbO_3_	39.59			
Sb_2_O_4_	18.27	1250	19	body centered cubic + pyrochlore + sodium fluoride
NaF	42.14			
NaSbO_3_	69.05			
Sb_2_O_4_	2.90	1250	19	body centered cubic + trace sodium fluoride
NaF	28.05			
NaSbO_3_	49.28			
Sb_2_O_4_	11.37	1250	19	pyrochlore + body centered cubic + sodium fluoride
NaF	39.35			
NaSbO_3_	31.20			
Sb_2_O_4_	28.87	1250	19	pyrochlore + sodium fluoride
NaF	39.93			
NaSbO_3_	84.62			
Sb_2_O_4_	–	1268	19	ilmenite + cubic
NaF	15.38			
NaSbO_3_	74.42			
Sb_2_O_4_	2.32	1261	1	distorted cubic + ilmenite
NaF	23.26	1268	19	distorted cubic + NaF
NaSbO_3_	70.00			
Sb_2_O_4_	3.33	1264	1	cubic + ilmenite
NaF	26.67			
NaSbO_3_	65.96			
Sb_2_O_4_	4.26	1266	1	cubic + ilmenite
NaF	29.78	1267	19	cubic + NaF
NaSbO_3_	62.96			
Sb_2_O_4_	4.94	1266	1	cubic + NaF
NaF	32.10	1267	19	cubic + NaF
NaSbO_3_	58.82			
Sb_2_O_4_	5.89	1267	19	cubic + NaF
NaF	35.29			
		1000	1	ilmenite + trace NaF
NaSbO_3_	68.00	1252	16	cubic + trace ilmenite
Sb_2_O_4_	4.00	1265	.1	cubic + NaF
NaF	28.00	1265	1.5	cubic + NaF
		1265	72	cubic + NaF

a/ Preheated at 750°C for 60 hours open.

## Footnotes

1 Figures in brackets indicate the literature references at the end of this paper.

2 The use of psi, bar, and kbar follows the current common practice of workers in the field. Note that 1 bar ≡ 10^5^ N/m^2^ (or pascal) ≡ 10^6^ dyn/cm^2^ ≡ 0.9869 atm ≡ 14.504 psi. The accepted international standard (SI) unit of pressure is the pascal or newton per meter squared.
